# Supplementary material for: Methods for Predicting Ethylene/Cyclic Olefin Copolymerization Rates Promoted by Single-Site Metallocene: Kinetics Is the Key
Source: Polymers (Basel). 2022 Jan 24;14(3):459. doi: 10.3390/polym14030459 (PMC8839136; doi:10.3390/polym14030459)
Supplement: Supplementary file 1 [file polymers-14-00459-s001.zip › polymers-1529673-supplementary.pdf]

Supporting information

Methods for Predicting Ethylene/cyclic Copolymerization Rates promoted by Single-sight Metallocene: Kinetics is the Key

Amjad Ali[1, 5], Ahmad Naveed[1], Tahir Rasheed[2]*, Tariq Aziz[3], Muhammad Imran[4], Zhang Ze Kun[5], Muhammad Wajid Ullah[6], Aziz Ur Rehman[4], Fan Zhiqiang[5], Li Guo[1]*

[1]Research School of Polymeric Materials, School of Materials Science & Engineering, Jiangsu University, Zhenjiang, 212013, P.R China.

[2]Interdisciplinary Research Center for Advanced Materials, King Fahd University of Petroleum and Minerals (KFUPM), Dhahran 31261, Saudi Arabia.

[3]Westlake University, School of Engineering Yunqi Campus, Hangzhou, Zhejiang 310024 PR China.

[4]MOE Key Laboratory of Macromolecular Synthesis and Functionalization, Department of Polymer Science and Engineering, Zhejiang University, Hangzhou, 310027, P.R. China.

[5]Department of Chemistry, Government College University, Lahore, Pakistan.

[6]Biofuels Institute, School of Environment and Safty Engineering, Jiangsu University, Zhenjiang, 212013, P.R China.

***Corresponding author:** E-mail address: masil@sjtu.edu.cn (T. Rasheed);

liguo@ujs.edu.cn (Li Guo).

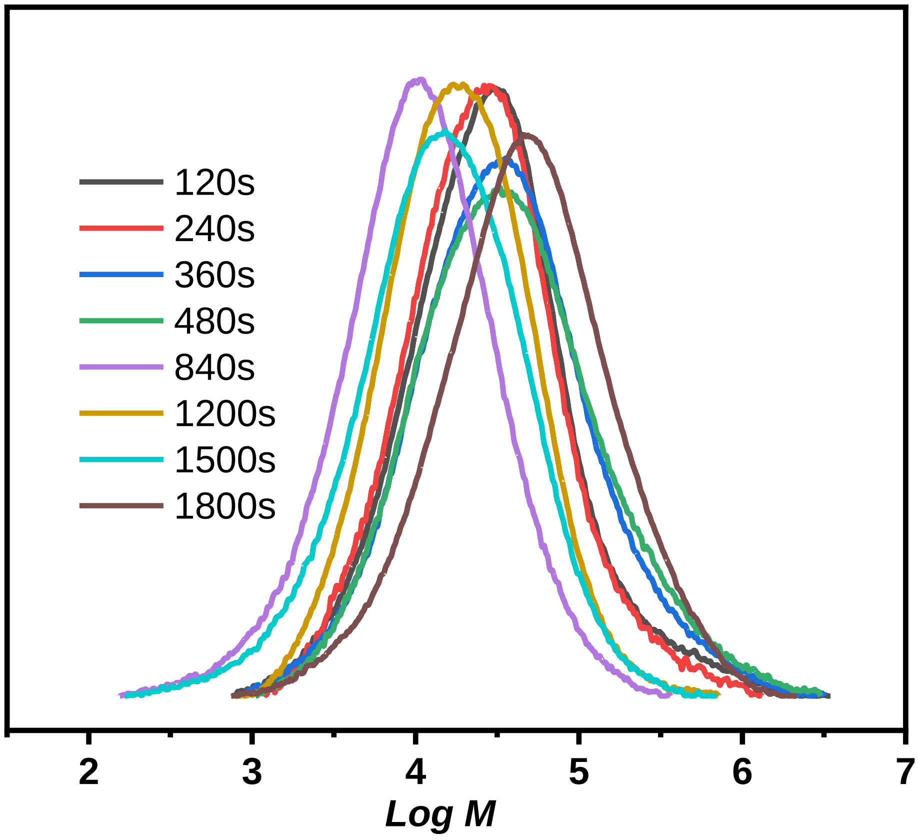

Figure S1. Change of molecular distribution of E/VCH copolymers with polymerization time

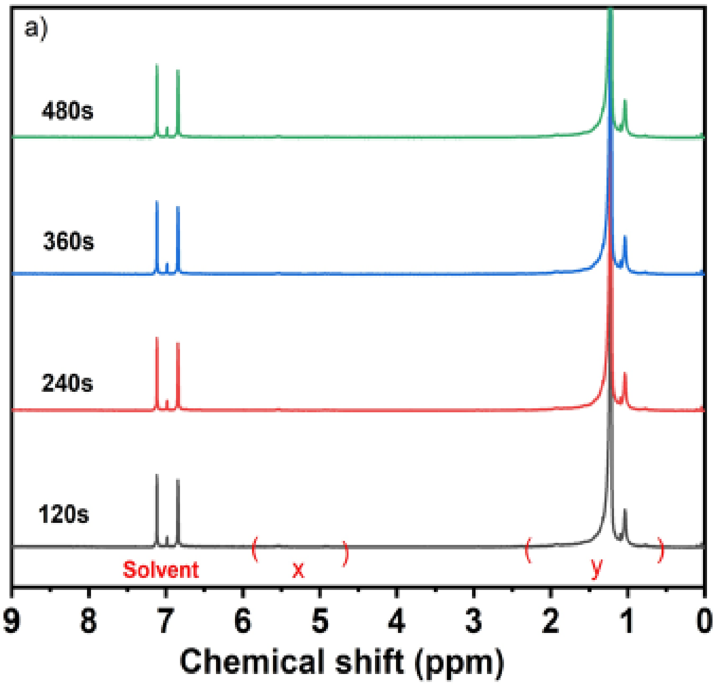
a)
480s
360s
240s
120s
Solvent
(x)
(y)
9 8 7 6 5 4 3 2 1 0
Chemical shift (ppm)

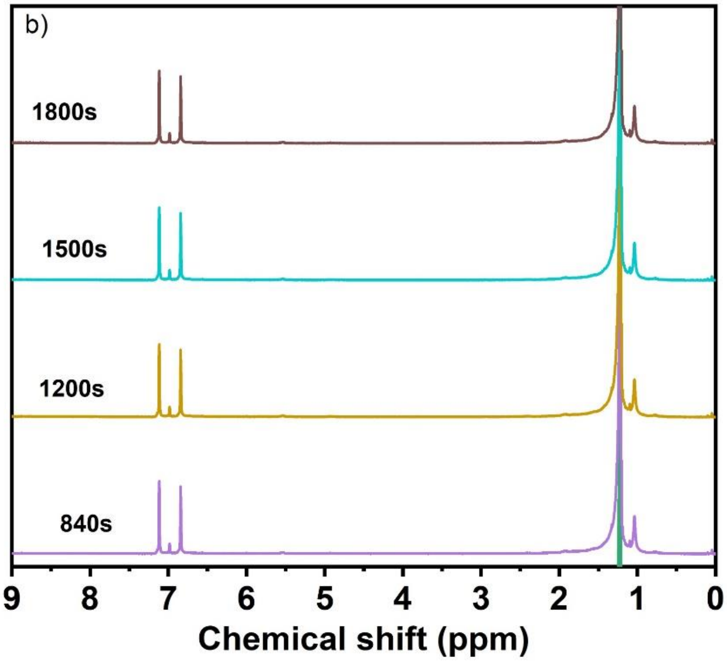
b)
1800s
1500s
1200s
840s
9 8 7 6 5 4 3 2 1 0
Chemical shift (ppm)

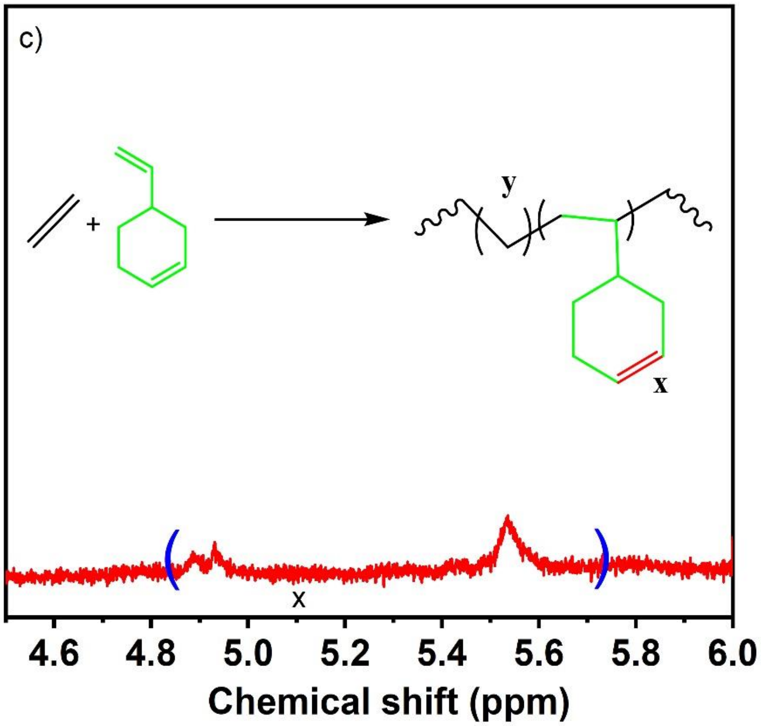

Figure S2 HNMR Spectra's of the E/VCH copolymer

Table S1 Calculation of R_P^{VCH} and K_P^{VCH} in the ethylene-VNB copolymerization.

RUN	Time<br>see	VCH In polymer	VCH in Pol wt%	Mol_{VCH}/mol_{cat}	R_P^{VCH}/mol_{cat}<br>molVNB/molcat s	S<br>Ppm	*C<br>%	[VCH]<br>mol/L	*kp* VCH<br>L/mol s
1.1	120	1.43	0.053	114	0.78	14.23	14.72	0.0572	93
1.2	240	1.56	0.058	243	0.66	12.63	25.68	0.0539	48
1.3	360	1.65	0.061	297	0.55	16.09	37.89	0.0526	28
1.4	480	1.53	0.057	331	0.46	19.97	49.16	0.0517	18.1
1.5	840	1.46	0.054	481	0.27	14.31	61.27	0.0480	9.18
1.6	1200	1.41	0.052	550	0.16	13.06	66.17	0.0463	5.228
1.7	1500	1.35	0.050	628	0.1	11.54	69.58	0.0443	3.244
1.8	1800	1.22	0.046	613	0.069	11.30	73.38	0.0447	2.1048

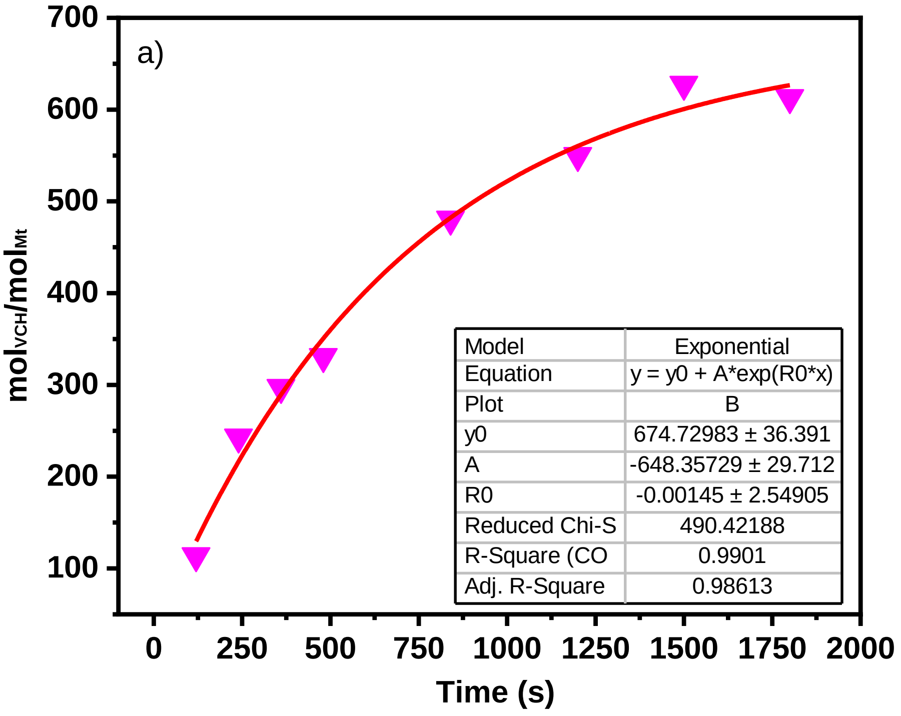

a)
Model | Exponential
Equation | y = y0 + A*exp(R0*x)
Plot | B
y0 | 674.72983 ± 36.391
A | -648.35729 ± 29.712
R0 | -0.00145 ± 2.54905
Reduced Chi-S | 490.42188
R-Square (CO | 0.9901
Adj. R-Square | 0.98613
mol_VCH/mol_Mt
Time (s)

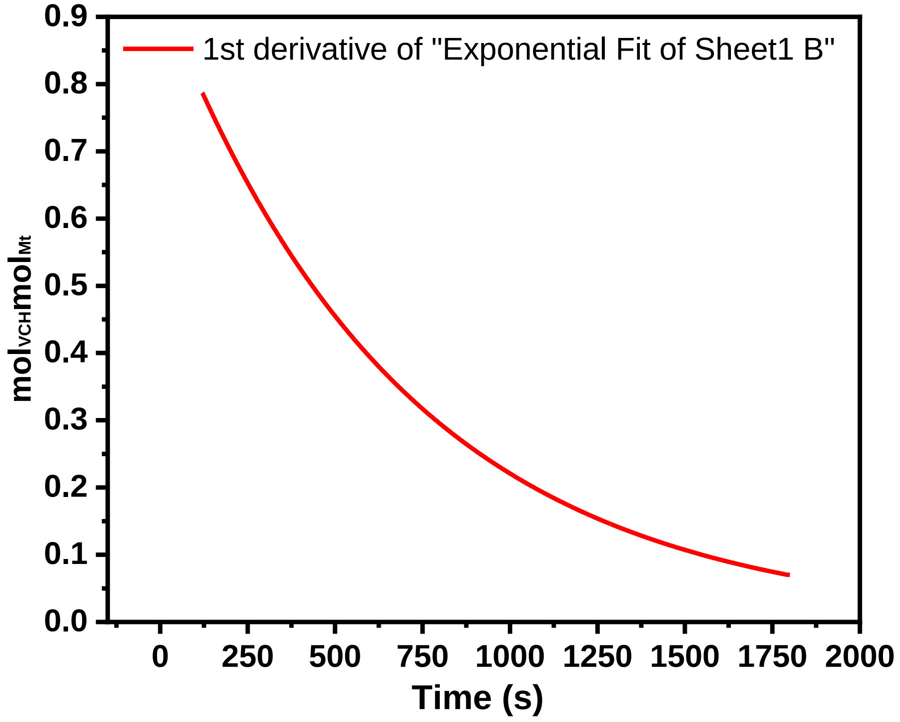

1st derivative of "Exponential Fit of Sheet1 B"
mol_VCH mol_Mt
Time (s)

Figure S3.The plot of mol_{VCH}/mol_{cat} vs. time, and Differentiate.

Table S2. Calculation of R_P^E and K_P^E in the ethylene-VCH copolymerization.

RUN	Time see	VCH In polymer	VCH In Pol wt%	S Ppm	*C %	mE g	mol_E/mol_{cat}	R_P^E molE/molcat s	*kp* E L/mol s
1.1	120	1.43	0.053	14.23	14.72	0.275	7846	39.63	6410
1.2	240	1.56	0.058	12.63	25.68	0.537	15347	36.72	3405
1.3	360	1.65	0.061	16.09	37.89	0.620	17710	33.94	2133
1.4	480	1.53	0.057	19.97	49.16	0.745	21294	31.41	1521
1.5	840	1.46	0.054	14.31	61.27	1.135	32431	24.86	966
1.6	1200	1.41	0.052	13.06	66.17	1.346	38449	19.67	708
1.7	1500	1.35	0.050	11.54	69.58	1.605	45863	16.17	553
1.8	1800	1.22	0.046	11.30	73.38	1.737	49634	13.32	432

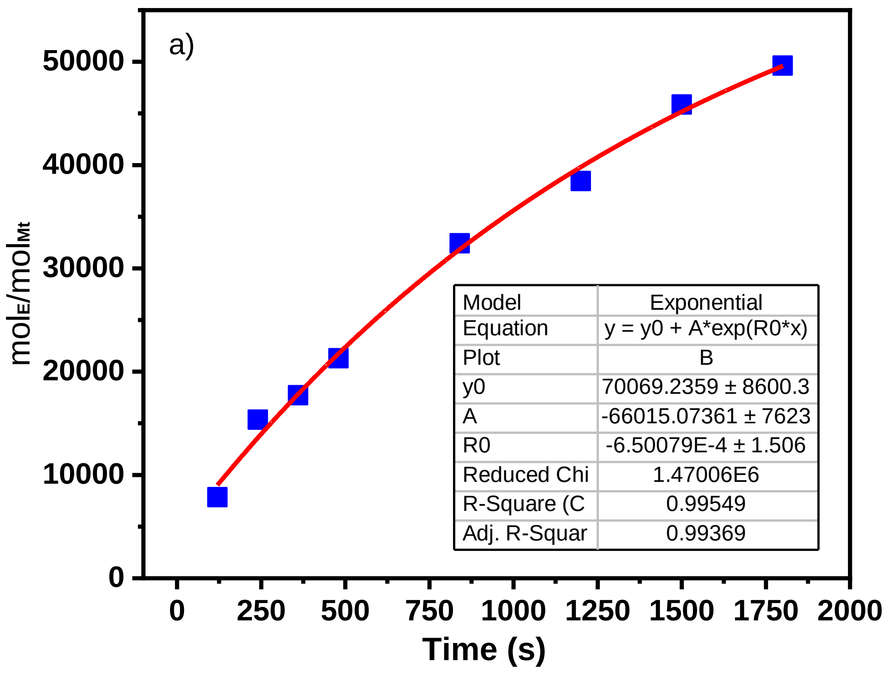

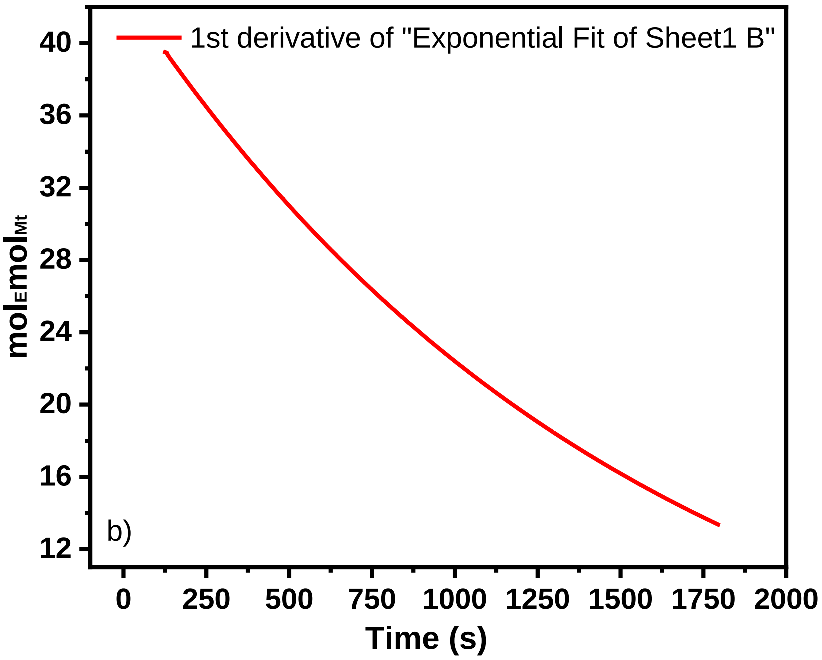

Figure S4.The plot of mol_E/mol_{cat} vs. time, and Differentiate
